# A Systematic Review on Non-mammalian Models in Epilepsy Research

**DOI:** 10.3389/fphar.2018.00655

**Published:** 2018-06-27

**Authors:** Muhammad Faiz Johan Arief, Brandon Kar Meng Choo, Jia Ling Yap, Yatinesh Kumari, Mohd Farooq Shaikh

**Affiliations:** ^1^MBBS Young Scholars Program, Jeffrey Cheah School of Medicine and Health Sciences, Monash University Malaysia, Bandar Sunway, Malaysia; ^2^Neuropharmacology Research Laboratory, Jeffrey Cheah School of Medicine and Health Sciences, Monash University Malaysia, Bandar Sunway, Malaysia; ^3^School of Science, Monash University Malaysia, Bandar Sunway, Malaysia

**Keywords:** epilepsy, fruit fly, drosophila, leech, planaria, roundworm, tadpole, zebrafish

## Abstract

Epilepsy is a common neurological disorder characterized by seizures which result in distinctive neurobiological and behavioral impairments. Not much is known about the causes of epilepsy, making it difficult to devise an effective cure for epilepsy. Moreover, clinical studies involving epileptogenesis and ictogenesis cannot be conducted in humans due to ethical reasons. As a result, animal models play a crucial role in the replication of epileptic seizures. In recent years, non-mammalian models have been given a primary focus in epilepsy research due to their advantages. This systematic review aims to summarize the importance of non-mammalian models in epilepsy research, such as in the screening of anti-convulsive compounds. The reason for this review is to integrate currently available information on the use and importance of non-mammalian models in epilepsy testing to aid in the planning of future studies as well as to provide an overview of the current state of this field. A PRISMA model was utilized and PubMed, Springer, ScienceDirect and SCOPUS were searched for articles published between January 2007 and November 2017. Fifty-one articles were finalized based on the inclusion/exclusion criteria and were discussed in this review. The results of this review demonstrated the current use of non-mammalian models in epilepsy research and reaffirmed their potential to supplement the typical rodent models of epilepsy in future research into both epileptogenesis and the treatment of epilepsy. This review also revealed a preference for zebrafish and fruit flies in lieu of other non-mammalian models, which is a shortcoming that should be corrected in future studies due to the great potential of these underutilized animal models.

## Introduction

Epilepsy is a type of central nervous system (CNS) disorder that affects neuronal activity in the brain, causing unprovoked seizures and other behavioral changes (Mussulini et al., 2013). An abnormality in neuronal activity relates to excessive electrical discharges which results in seizure episodes that can vary in frequency from less than one per year to a few times per day. Seizures can be classified into two main types, partial seizures and generalized primary seizures. Furthermore, a seizure is divided into three stages: aura, ictus and postictal. The aura stage is experienced by the victim when he or she begins to display abnormal sensations such as nausea, headaches, abnormal senses and sudden emotions (fear, panic, etc.). The victim then enters the ictus stage in which the symptoms can be convulsive (vigorous jerking of the body and loss of consciousness) or non-convulsive (inability to respond and muscle spasms), depending on the type of seizure. The postictal stage is also known as the recovery stage and is when the victim begins to experience post-seizure symptoms such as drowsiness, confusion and partial paralysis. The onset of this disorder can occur in all ages, although it mostly occurs in childhood or later adulthood and is the fourth most common CNS disorder (England et al., 2012).

Currently around 70 million of the world's population is affected by epilepsy (Cunliffe et al., 2015). Epilepsies with known causes (secondary epilepsies) may be the result of prenatal brain damage, genetic abnormalities which lead to brain deformities, severe brain trauma, severe stroke, infection of the brain and brain tumors. Sufferers tend to have problems both physical (bruising and fractures from seizure) (Baker et al., 1997) and psychological (anxiety and depression) (Ettinger et al., 1998) in nature. The abnormal epileptic brain waves typical of epilepsy patients can be detected using the magnetoencephalography (MEG) and electroencephalogram (EEG) techniques (Karis, 2008). Unfortunately, epilepsy cannot be cured and can only be symptomatically treated. Treatment typically involves the usage of anti-epileptic drugs (AEDs) (White et al., 2007), surgery (Jette et al., 2014), vagus nerve stimulation (Uthman et al., 1993) or the ketogenic diet (Neal et al., 2008). However, about a third of the epileptic population do not respond to current epilepsy treatments (White et al., 2007). Moreover, epileptic individuals face a lower quality of life as they are burdened by the limitations placed on their physical activities and can be subjected to prejudice due to their disruptive seizures (Baker et al., 1997).

The usage of animal models is essential for the study of epilepsy as the process of epileptogenesis and ictogenesis cannot be induced in human trials due to ethical reasons. Rats and mice have previously been used for animal testing due to their similar morphological structure to humans. However, in recent years, non-mammalian epilepsy models have been the primary focus in animal testing due to multiple factors. These factors including increased cost-effectiveness, high genetic correlation with humans and rapid breeding, all of which improves the efficiency of epilepsy research (Cunliffe et al., 2015). In non-mammalian epilepsy models, Pentylenetetrazole (PTZ) (Kundap et al., 2017), kainic acid (KA) (Kandratavicius et al., 2014) and pilocarpine (PILO) (Kandratavicius et al., 2014) are among the proconvulsants used for stimulating seizures. In addition, electrical stimulation may also be used to induce seizures in animal models and differs from proconvulsants in that its effects can be studied without the continued presence of the epileptogenic cause (Kandratavicius et al., 2014). Thus, a comprehensive literature research was commenced to establish a systematic review which discusses the importance and usage of non-mammalian models in studies concerning epilepsy. This review aims to consolidate current information on the use and importance of non-mammalian models in epilepsy testing to better aid the planning of future research and screening of potential anticonvulsive compounds.

## Materials and methods

### Search method

Studies between January 2007 and November 2017 were considered for evaluation. This restriction is to ensure that the most recent publications are covered in this review while minimizing the possibility of inadvertently excluding older studies. An initial search of relevant studies was performed using Google Scholar to grasp the general scope of the topic. After that, a final search of relevant studies was conducted using several databases which were: PubMed, SCOPUS, SpringerLink and ScienceDirect. A search using the keywords “epilepsy,” “seizures,” “animal model,” and “convulsions” was first done to generate a list of relevant articles for each database. After that, the keywords “non-mammalian,” “drosophila,” “leech,” “planaria,” “roundworm,” “tadpole,” and “zebrafish” were searched for in each of the generated lists and used to select the final articles. This was done by first screening through the abstracts of the generated results, before proceeding to full text screening of potentially highly relevant articles.

### Study selection and inclusion criteria

Studies that were searched and considered for the systematic review were limited to original research articles as other publications (symposiums, conferences, editorials, book chapters, reviews and systematic reviews) would not provide sufficient information for evaluation and comparison. Any duplicated articles from the different databases were removed and articles that have no relevance to the importance of non-mammalian models for epilepsy research were also excluded. The selection of studies was conducted as per PRISMA guidelines (Moher et al., 2015).

## Results

**Figure 1:**
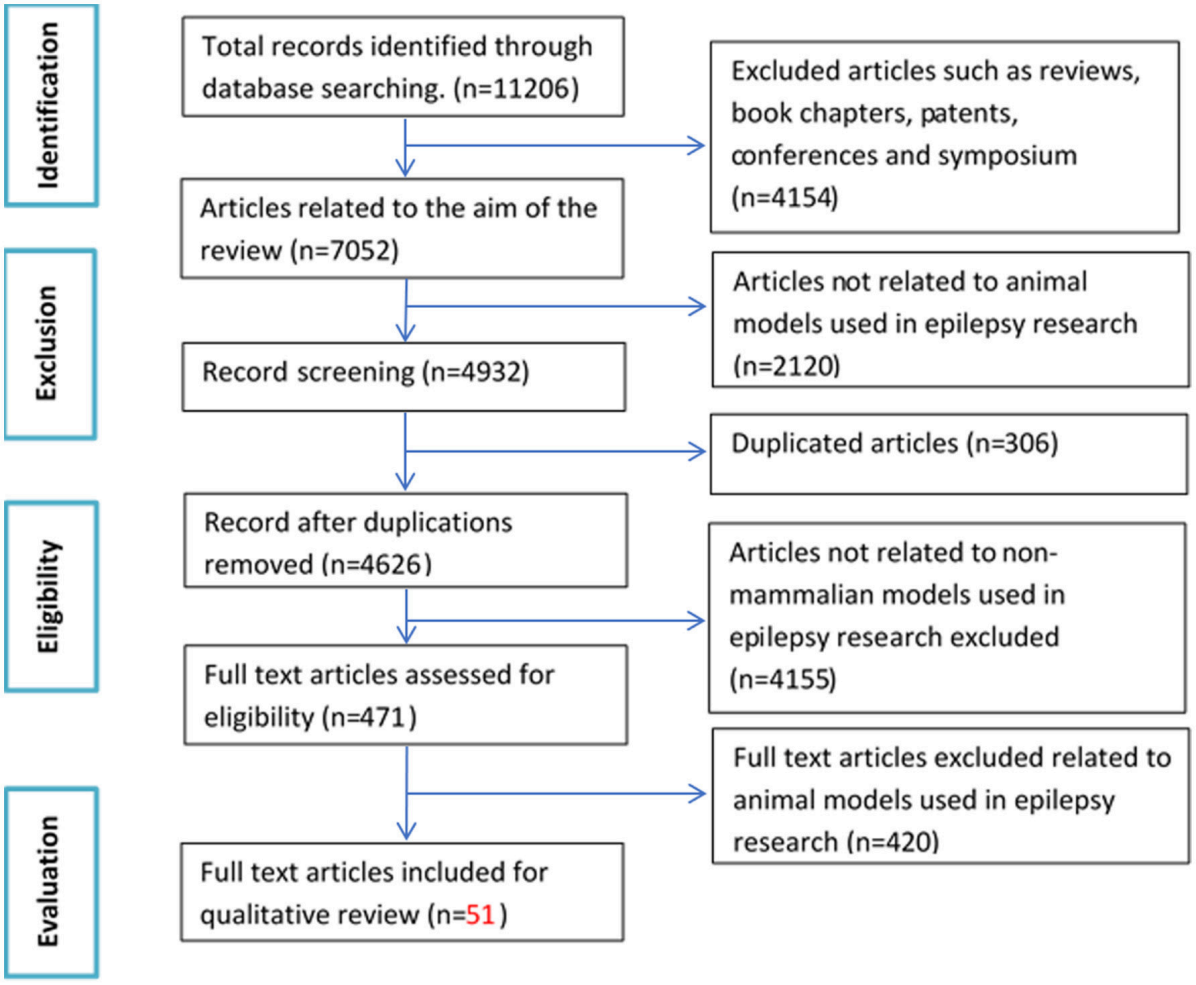
Flow chart of study selection criteria based on Preferred Reporting Items for Systematic Reviews and Meta-Analyses (PRISMA) guidelines.

**Figure 2:**
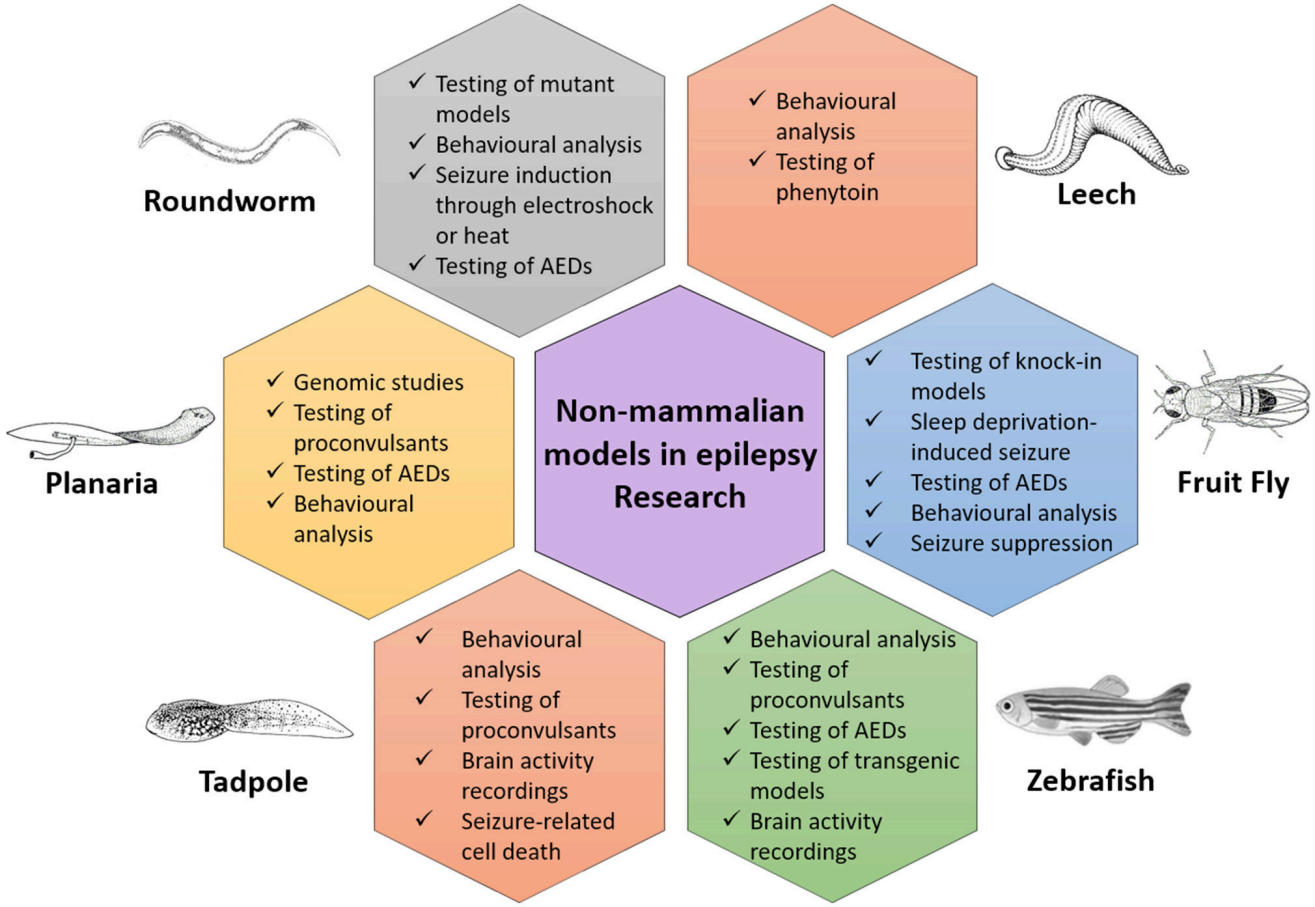
A brief overview of all the non-mammalian models for epilepsy research in this review.

The initial search based on the keywords mentioned in the methodology yielded a total of 11,206 records. After applying exclusion criteria, total articles removed were 11,155, which includes; (a) 4,154 non-original research articles (b) 2,120 articles not related to animal models used in epilepsy research, (c) 306 duplicates, (d) 4,155 articles not related to non-mammalian models used in epilepsy research and (e) 420 full text articles not relevant to the aim of the review (Figure 1). Fifty-one eligible articles were included, compiled in Table 1 and discussed in the present systematic review. Based on the inclusion criteria, the final articles selected for evaluation consist of 19 articles relating to fruit flies, one article relating to leeches, three articles relating to planaria, two articles relating to roundworms, two articles relating to tadpoles and 24 articles relating to zebrafish, for a grand total of 51 articles. A brief overview of all the non-mammalian models for epilepsy research in our review is summarized in graphical form in Figure 2.

**Table 1:** Summary of the selected articles, together with high throughput screening feasibility.

**Animal Model *Scientific name* (*n* = Sample size)**	**Strain**	**Epileptogenic insult type (parameters)**	**Experimental findings**	**Possibility of high-throughput screening (HTS)**	**References**
Fruitfly *Drosophila melanogaster* (*n* = 5–100)	GEFS+ *SCN1A* mutation	•High temperature (40°C) •PTZ (0–0.4 mM)	•GEFS+ flies exhibit a heat-induced seizure phenotype •Blockade of GABA receptors increases sensitivity to heat-induced seizures in GEFS+ flies	Yes (Stilwell et al., 2006)	Sun et al., 2012
	Canton Special (CS)	High frequency stimulation (0.5 ms pulses at 200 Hz for 300 ms)	•Acute feeding of potassium bromide ameliorates *bss* phenotypes •Acute feeding of Top1 inhibitors does not eliminate seizure-like behaviors, but did reduce the mean recovery time (MRT) of BS mutants		Song et al., 2008
	*para^*bss*1^*	High-frequency electrical brain stimulation (0.5-ms pulses at 300 Hz for 400 ms)	•Recovery time from BS paralysis for *para^*bss*1^* varies with genetic background, age, and other factors •Recovery time appears to be primarily dependent on the number of bouts of tonic–clonic like activity		Howlett et al., 2013
	•Easily shocked *(eas)* •Bang senseless *(bss)* •Technical knockout *(tko)*	•70 ml fly vials •VWR VortexGenie (10 s)	•The *atu* mutation reduces SLA and shortens the recovery time in BS mutants		Stone et al., 2013
	*Drosophila melanogaster*	•Fly vials (10 s) •39°C water bath •C4162 High Power Strobe, Chaney Electronics (10 Hz for 10 s)	•Glia endogenously express both Dube3a and ATPα proteins •Overexpression of Dube3a in glia using repo-GAL4 results in seizure susceptibility •Glial Dube3a overexpression alters neuronal architecture •Glial-specific overexpression of Dube3a causes reduced intercellular K^+^ in glial cells		Hope et al., 2017
	Wild type Oregon-R	PTZ (3.48, 0.33, and 5 mg/ml)	•Convulsions were not observed, visual examination found hyperkinetic behavior in PTZ treated flies •NaVP and LEV alleviates PTZ induced climbing speed deficit in flies •LEV exerted a long-term effect		Mohammad et al., 2009
	pk*^*sple*1^*/pk*^*sple*1^*	Fly vials (20 s followed by 10 s)	•Drosophila with homozygous prickle mutations display seizures		Tao et al., 2011
	•Easily shocked*(eas)* •Bang senseless *(bss)* •Technical knockout *(tko)*	70 ml fly vials (10 s)	•Acute exposure (10 s) to 100% CO^2^ caused SLA in all three BS mutants •Refractory period following acute gas exposure •Susceptibility to SLA and anaesthetization following hypercapnia •Ability of hypoxia to trigger SLA •Prolonged exposure to anesthetic gases delayed SLA		Whelan et al., 2010
	•Canton-S (wild-type) •Technical knockout *(tko)* •sesB •eas^alaE13^ BS mutants	Enclosed square recording arena with Vortex Genie2 (10 s)	•The ketone body β-hydroxybutyrate (β-HB) reduces SLA in the *eas* BS strain •The anticonvulsant effects of β-HB on SLA are partially mediated by both K_ATP_ channels and GABA_B_ signaling		Li et al., 2017
	Wild type Canton S	PTX (5 μM) 4-aminopyridine (3 mM)	•Exposure to proconsulvants heightens stimulation of neuronal activity •Exposure to Gbp reduces synchronicity		Streit et al., 2016
	•Bang senseless •W^1118^ •elav-Gal4 •*UAS-MRP1* •*UAS-MRP1: elav-Gal4*	Fly vials that were vortexed at VORTEX-5 for 10 s	•PHT reduces mean recovery time of three control groups containing the *bss* mutation •PHT and VPA caused negative effects on eclosion rate but positive effect on seizure behavior		Bao et al., 2011
	Wild-type	High-frequency stimulation 0.5 ms pulses at 200 Hz for 300 ms using oscilloscope Minimal voltage: 2 V	•During induction of seizure, oscilloscope shows seizure-like activity •Presence of 7 min refractory period in flies after high frequency stimulation •If seizure is still induced, refractory period increases to 17 min		Howlett and Tanouye, 2009
	•Wild-type •para^bss1^ •*eas* •*sda*	High-frequency stimulation 0.5-ms pulses at 200 Hz for 400 ms using electrodes	•Injection of saline control caused a slight increase in seizure threshold whereas injection of 25 mM valproate saline solution caused a significant increase •Seizure sensitivity of para^bss1^ is the most difficult to suppress		Howlett and Tanouye, 2013
	•*eas^*PC*80^* •*sda ^*iso*70.8^*	High-frequency electrical brain stimulation (0.4-msec pulses at 200 Hz for 300 ms using electrodes)	•sh^its1^ mutation in flies contributes to suppressing seizures •Seizure-like activity is related to the synaptic depletion •Disruption of synaptic transmission in excitatory neurons results in action potentials that are linked with seizures		Kroll et al., 2015
	•*Stress sensitive B (sesB9^*Ed*−4^)* •bss paralytic mutant with loss of ANT •*Seizure (sei^*ts*^)* •Temperature-sensitive paralytic mutant •UAS-*sesB*^31320^ •UAS-*sesB^36661^*	Sleep deprivation Fly vials (10 s) and vortex machine	•Sleep deprivation can further influence seizure activity when *sesB* is disrupted •VPA can decrease susceptibility to sleep deprivation-enhanced seizure in *sesB9^*Ed*−4^* •The effect of sleep deprivation on seizure activity can be observed using mechanical stimuli •In early development, sleep deprivation can increase susceptibility to sleep deprivation-enhanced seizure		Lucey et al., 2015
	•Canton-S wild-type •Cry^03^homozygotes^29^ •Larvae	Electroshock (10–30 V) Direct current pulse, created	•Exposure to pulsed blue light increased seizure duration •100 mT magnetic field increased the effect of blue light on seizure severity and is light dependent •Antiepileptic drugs can prevent prolongation of seizure due to their effect on neuronal activity		Marley et al., 2014
	•Canton-S wild-type •Bang senseless •eas	High-frequency electrical brain stimulation (0.5-ms pulses at 200 Hz for 300 ms)	•Mechanical shock results in six specific seizure phases in flies •Genetic background can influence seizure duration •bss flies have lower seizure threshold •The behavior of *bss* mutants is similar to other BS mutants such as *eas^*PC*80^, sda^*iso*7.8^*, and *tko^25*t*^*		Parker et al., 2011
	•*para^*bss*1^* •*eas^*PC*80^* •*sda ^*iso*7.8^*	High frequency brain stimulation (0.5 ms-pulses at 200 Hz for 300 ms using tungsten electrodes)	•*cac^*TS*2^* acts as a general seizure-suppressor mutation and can revert the effects of mutations such as sda, eas and para^bss1^ •*cac^*TS*2^* is a seizure-resistant mutant at room temperature but a seizure-sensitive mutant at high temperature		Saras and Tanouye, 2016
	•*eas*^PC80^ •*sda ^*iso*7.8^* •*topo I*	High frequency brain stimulation (0.5 ms-pulses at 200 Hz for 300 ms using tungsten electrodes)	•*top1^*JS*^* mutation is a general seizure-suppressor •*top1^*JS*^* mutation increases seizure thresholds and reduces the recovery time of *eas* flies		Song et al., 2007
Medicinal leech *Hirudo verbana*	Stage IV larvae (*cca-1 mutant*)	PTZ (4–10 mM)	No seizures in T-type Ca^2+^ channel (*cca-1*) mutant worms	No data found	Hahn and Burrell, 2015
Planaria (*n* = 8–20)	Brown *D. tigrina*	NMDA (1, 3, 10 mM) PTX (0.01–5.0 mM) Nicotine (0.1–10 μM)	Planaria exhibit an increasing number of sudden asynchronous convulsive movements in a dose dependent manner when exposed to proconvulsants	No data found	Ramakrishnan and DeSaer, 2011
	Brown *D. tigrina*	Glutamate (0.6 mM) NMDA(1.4 mM) Semicarbazide (4.5 mM).	•Riluzole reverses I-glutamate, NMDA and semicarbazide induced PSLA •(+)-MK-801 reverses I-glutamate and NMDA induced PSLA		Ramakrishnan et al., 2013
	*Dugesia dorotocephala*	NMDA (0.01, 0.1, 1, 3, 10 mM) or water	•NMDA produced PSLA •MK-801 or DNQX antagonizes NMDA-induced PSLA •Topiramate antagonizes PSLA induced by NMDA or AMPA		Rawls et al., 2009
Roundworm *Caenorhabditis elegans* (*n* = 4–6)	Stage IV larvae (wild type N2)	High temperature (26°C-28 ± 1°C)	Seizure frequency was significantly decreased by Baccoside A	Yes (O'reilly et al., 2014)	Pandey et al., 2010
	Stage IV larvae (*cca-1 mutant*)		No seizures in T-type Ca^2+^ channel (*cca-1*) mutants		
	•Bristol N2 •CB156 *unc-25(e156)* •CB382 *unc-49 (e382)*	Electrical shock (200 Hz, 3.5 ms, 47 V) PTZ (72.0 mM)	Electric shock induces paralysis and convulsions in *C. elegans*		Risley et al., 2016
Tadpole *Xenopus laevis*	Albino *Xenopus laevis* (*n* = 20)	PTZ (15 mM) Bicuculline Picrotoxin Kainic acid (0.25 mM) Pilocarpine (75 mM) 4-ami-nopyridine, 4-AP (1 mM)	•All proconvulsants caused seizures which are divided into classes; (I) Rapid swimming, (II) Behavior arrest, (III) Loss of posture, (IV) Repetitive side-to-side lateral movement of head, (V) Fast, alternating contractions of axial musculature •A low number of TUNEL-positive and PI-positive cells were present, indicating progressive cell-loss within normal brain growth	No data found	Hewapathirane et al., 2008
	Wild type	PTZ (10 mM or 15 mM)	•PTZ induces seizures in tadpoles, causing uncontrolled tail bends and excessive turning •Seizure susceptibility decreases when the level of putrescine increases •Endogenous protective mechanisms prevent long-term cell damage due to the presence of polyamine		Bell et al., 2011
Zebrafish *Danio rerio*	Larvae, 7 d.p.f, Ekkwill strain	PTZ (40 mM)	•PTZ induces agitation (Stage I) before degrading into occasional body-stiffening and loss of posture (Stages II and III) •TPR, VPA, LTG •reduces PTZ-induced movement as to compare with VHC+PTZ, within 30 min. •GBP increases PTZ-induced movement •Other AEDs were inactive at their MTC	Yes - embryo (Liu et al., 2012)	Afrikanova et al., 2013
	•Adult, wild type (*n* = 6)	PTZ (220 mg/kg)	•The PTZ-treated group had an increase in distance, velocity, mobility, and circular rotations •GBP prevented PTZ-induced increases in zebrafish •cephalic field potential		Banote et al., 2013
	Larvae (*n* = 5)	PTZ (15 mM)	•SC-560 reduces *c-fos* mRNA expression compared to PTZ group whereas SC-236 has no effect •SC-560 prevents PTZ-induced increase of locomotor activity whereas SC-236 had no effect		Barbalho et al., 2016
	Larvae, WIK wild-type (*n* = 12)	PTZ (20 mM)	Oxcarbazepine, zonisamide and diazepam caused a decrease in locomotor activity		Berghmans et al., 2007
	•Adult, (AB), wild-type strain •Male (*n* = 8; 10 months) •Female (*n* = 9; 8 months)	PTZ (15 mM)	•Zebrafish experienced 4.93 seizure events on average •Epileptiform discharges last for 85 s on average		Cho et al., 2017
	Adult, WIK strain	PTZ (15 mM)	•Reactive gliosis manifests after PTZ-induced seizures •Total number of leukocytes increases after seizure •Increased cellular proliferation in ventricular zone and parenchyma		Duy et al., 2017
	Adult, wild-type (4–5 months)	PTZ (2, 4, 6, and 8 mM) Caffeine (1, 10, or 30 μM)	•Lower concentration (2 mM) of PTZ evokes stage I (increased swim activity) •Higher concentrations (4, 6 and 8 mM) of PTZ evokes stages II and III (II—rapid whirlpool-like circling swim, III—clonus-like seizures) •Valproic acid, gabapentin, lacosamide and carbamazepine increases latency to all stages •Pregabalin was ineffective		Gupta et al., 2014
	Larvae, 7 d.p.f	PTZ (15 mM) Picrotoxin Biculline	s334/Colbert mutant demonstrates inability to generate long duration epileptiform discharges in response to PTZ		Hortopan et al., 2010b
	•Wild type larvae, 3 d.p.f •Wild-type (*n* = 22), mib mutant (*n* = 28)	PTZ (15 mM)	•Stage 1 and 3 seizures were noted in mib^hi904^ mutants •93% of mib^hi904^ mutants acquire recurrent spontaneous multi-spike bursts > 1,000 ms in duration		Hortopan et al., 2010a
	•7 d.p.f., Larvae, wild-type (AB) •5 d.p.f morpholino-injected larvae (WT) •7 d.p.f scn1La mutant larvae (HO)	PTZ (40 mM)	•PTZ-treated larvae suffered more seizures than VHC-treated larvae •PTZ-treated larvae had a longer seizure duration than VHC-treated larvae •Seizures per larvae in the HO group is higher than the WT group •Chemical models produced frequent and long seizures as to compared to genetic models		Hunyadi et al., 2017
	Adult (3–4 months old) heterogenous WT stock	PTZ (170 mg/kg)	•PTZ group had seizures characterized by abnormal circular movements •PHY, RSV, OXC, GBP, and DZP provided resistance against PTZ		Kundap et al., 2017
	•Larvae (*n* = 10) •Embryo	Ginkgotoxin (40 mM)	Ginkgotoxin exposure induces seizure-like behavior within embryos		Lee et al., 2012
	Larvae (7, 15, and 30 d.p.f.)	Kainic acid (100, 300, and 500 μM)	•No locomotor or seizure activity recorded in 7 d.p.f. larvae •Locomotor activity detected in 15 d.p.f larvae •No locomotor or seizure activity recorded in 30 d.p.f. larvae		Menezes et al., 2014
	Adult, 4–6 months (*n* = 12)	PTZ (5, 7.5, 10, and 15 mM)	•Zebrafish immersed in PTZ solution experienced seizures •Seizure scores of zebrafish increases as PTZ concentration increases		Mussulini et al., 2013
	•Adult, wild-type *TupLF* strain •Adult, transgenic Tg[*HuC:GFP*]	Loss of cln3 protein using a knockout model	•Using *in situ* hybridization, cln3 gene expression was shown in WT zebrafish •EEG reveals increased frequency activity and higher amplitude in cln3 ATG MO morphants, which reflects epileptiform activity •Loss of cln3 protein in zebrafish causes motor abnormalities		Packer et al., 2016
	•Larvae, wild-type 7–14 d.p.f. (*n* = 5) •*Danio rerio* larvae, aldh7a1 mutants 7–14 d.p.f. (*n* = 5)	Transgenic model aldh7a1	•Light induces rapid “whirlpool-like” swimming (stage 2) and body convulsions (stage 3) when exposed to aldh7a1 mutants at 10/11 d.p.f. •High number of bursts of abnormal electrical discharge with long duration and high amplitude was observed in the tested mutants •Pyridoxine treatment prevents seizure-like behavior		Pena et al., 2017
	•Adult, laboratory strain WIK •(9–14 months)	PTZ (15 mM)	•PTZ induces seizures within zebrafish •Eugenol at high concentration prolongs seizure latency •Visual appearance of seizure tracings was observed and can be differentiated into baseline, pre-seizure, seizure and post-seizure		Pineda et al., 2011
	•Adult, Female laboratory strain WIK •(9–14 months)	PTZ (15 mM)	•Hindbrain stimulation in •the locus coerulus region promotes seizure resistance •Higher PTZ concentration causes higher stimulation rate		Pineda et al., 2013
	Adult, wild-type strain	PTZ (7.5 mM)	•PTZ induces behavioral changes in zebrafish (Stage I, II, and III seizures) •Increased latency to clonus-like convulsions are due to pretreatments PHT, GBP, and VPA •PTZ has no effect on ATP, ADP, and AMP hydrolysis but increases ecto-ADA and soluble-ADA activities		Siebel et al., 2013
	Adult, AB strain (6–9 months)	Kainic acid (6 mg/kg)	•KA induces behavioral changes in zebrafish, followed by dose-dependent seizures •DHA supplemented groups showed an increase in latency at time of seizure onset		Sierra et al., 2012
	•Larvae, wild-type strain (*n* = 6) •Larvae, Scn1a mutant (*n* = 6)	Transgenic model with Scn1a mutation	•FA reduces epileptiform brain activity •σ1-agonist, 5-HT1D–or 5-HT2C-antagonist can prevent FA's effect on locomotor activity •66% decrease in monoamines due to FA treatment		Sourbron et al., 2017
	Larvae, AB wild-type	PTZ (1–25 mM)	•Stage II seizures were evoked in larvae •DA treatment reduces time in seizure latency •The DA cohort travel 5 times more than another other PTZ groups •DA treatment increases mobility of larvae		Tiedeken and Ramsdell, 2007
	Larvae, Tg (elavl3:GCamP6s), 4 d.p.f	PTZ (1–15 mM)	•Tetrodotoxin suppressed neuronal seizure activity caused by PTZ •GCaMP measurements can monitor basal activity and dynamics due to drug induced seizures •Valproate reduced larva motility and fluorescence		Turrini et al., 2017
	Larvae, AB wild-type	PTZ (1–16 mM) Picrotoxin, PTX (1–625 micrometer)	PTX-treated group has a higher maximum and lower minimum locomotor activity as compared with PTZ-treated group in dark conditions		Yang et al., 2017

*PTZ, Pentylenetetrazol; GEFS+, Generalized epilepsy with febrile seizures plus; GABA, γ-Aminobutyric acid; BS, Bang Sensitive; LEV, Levetiracetam; SLA, Seizure Like Activity; PTX, Picrotoxin; PHT, Phenytoin; VPA, Valproic Acid; Eas, Easily Shocked; Bss, Bang Sensless; Tko, Technical Knockout; Sda, Slam Dance; NMDA, N-Methyl-D-aspartic acid; PLSA, Planarian Seizure Like Activity; TPR, Topiramate; LTG, Lamotrigine; VHC, Vehicle Control; AED, Anti-Epileptic Drugs; MTC, Maximum Tolerated Concentration; GBP, Gabapentin; OXC, Oxcarbazepin; RSV, Rivastigmine; DZP, Diazepam; D.p.f, Days Post Fertilization; WT, Wild Type; ADA, Adenosine deaminase; KA, Kainic Acid; DHA, Docosahexaenoic acid; FA, Fenfluramine; DA, Domoic Acid*.

### Fruit fly (*Drosophila melanogaster*)

*Drosophila* have been widely used as a genetically tractable animal model for epilepsy research, especially the transgenic and mutant types. Seizures in *Drosophila* are defined as a period of brief leg twitches and is followed by a failure to maintain standing posture with leg shaking, abdominal muscle contractions, wing flapping and scissoring as well as proboscis extensions (Sun et al., 2012).

The usage of fruit fly models has been observed in 19 studies relating to epilepsy that were covered in this review.

#### Sleep deprivation-induced seizures

Sleep and seizures have already been associated in humans as sleep deprivation is known to cause paroxysmal epileptiform activity (Lucey et al., 2015), though the exact mechanisms which relate the two together are currently unknown. *Drosophila melanogaster* is a powerful model organism that has been successfully used to study human physiologic processes such as sleep and disease states such as epilepsy (Shaw et al., 2000). Research into sleep and the fruit fly has shown that not only do flies sleep, but that data obtained in flies can be applied directly to humans (Lucey et al., 2015) as biomarkers of sleepiness first identified in the fruit fly have also been found to be elevated in healthy humans after a prolonged period of wakefulness (Seugnet et al., 2006). *Drosophila* mutants that are prone to mechanical and temperature induced seizures are a validated model to investigate the molecular and cellular networks responsible for seizure phenotypes (Burg and Wu, 2012) as mechanical and temperature stress-induced seizures exhibit several similar features to seizures in humans, such as a stereotyped behavioral sequence of spasm-and-paralysis, followed by a refractory period when the mutant flies are no longer sensitive to their respective stress disturbance (Lucey et al., 2015). The effects of sleep deprivation on seizure activity are not confined to a particular class of seizure mutant and can be observed using mechanical stimuli or changes in temperature to induce seizures (Lucey et al., 2015).

#### Effect of cryptochrome-dependent magnetic field on seizure response in *Drosophila* larvae

*Drosophila* embryos exposed to pulsed blue light (470 nm) develop a heightened seizure-phenotype when tested post-embryonically at the third instar larvae stage, with the effect significantly potentiated when a magnetic field is also present (Marley et al., 2014). This is because the flavin adenine dinucleotide (FAD)-containing, circadian clock photoreceptor protein, cryptochrome (DmCRY) present in *Drosophila* central neurons, will render those neurons photosensitive and result in the increase in action potentials generated when illuminated with blue light (Fogle et al., 2011). The reason that magnetic fields potentiate the effect of blue light is that DmCRY plays a role in the magnetic sense of *Drosophila* (Gegear et al., 2010) and thus would also modulate the ability of light activated DmCRY to influence level of synaptic excitation in the *Drosophila* CNS.

#### Modeling epilepsy using *Drosophila* mutants

Recent technical advances have now made it practical to readily target and replace endogenous sequences in the fly genome using homologous recombination (Rong and Golic, 2000; Rong et al., 2002; Staber et al., 2011). Knocking-in of specific disease-causing mutations into the fly genome could provide a rapid and low-cost platform for studying the cellular mechanisms of human diseases. Knock-in flies can be used in combination with forward genetic screens to identify suppressor and/or enhancer mutations, a strategy that is challenging in humans and rodent models but well established in *Drosophila* (Song et al., 2007, 2008). One example is the human *SCN1A* sodium channel gene, which has more than 600 possible mutations which can confer a wide spectrum of epilepsies (Claes et al., 2009; Lossin, 2009; Catterall et al., 2010). *SCN1A* has more than 40 missense mutations that are known to be linked to an epilepsy syndrome termed “genetic epilepsy with febrile seizures plus” (GEFS+) (Sun et al., 2012).

While undoubtedly there are many mutant strains of *Drosophila* useful for the modeling of epilepsy that have been and will be discovered, the bang-sensitive (BS) group of *Drosophila* mutants are perhaps more popular than others. BS flies are useful for modeling epilepsy as they are significantly more prone to seizure like activity from a variety of insults as compared to their wild type counterparts (Burns et al., 2004; Stone et al., 2013). In addition, the seizure like activity displayed by BS flies such as violent bursts of uncoordinated leg, wings and abdomen contractions that are interspersed with paralysis, have parallels to human epilepsy (Lee and Wu, 2002; Stone et al., 2013). This bodes well for the validity of the BS model of epilepsy and any subsequent translation of results to human studies.

Prickle proteins, which were first discovered in *Drosophila*, are highly conserved (Spencer, 1945). *pk*^*sple1*^ mutants can be produced by crossing *pk*^*sple1*^ homozygous mutants with *yw*^*67*^ control flies. *pk*^*sple1*^ heterozygotes display none of the morphological abnormalities of the homozygotes but are significantly more bang sensitive than control flies of the same age. An epilepsy phenotype that is found in disparate species and crosses the invertebrate-vertebrate transition strongly suggests that the prickle proteins are part of a highly conserved evolutionary pathway for regulating seizures (Tao et al., 2011).

#### Brain electrical stimulation-induced seizures

As commonly seen in other models of epilepsy, electrical stimulation can also induce seizures in *Drosophila* (Song et al., 2007). This method essentially involves inserting electrodes into the *Drosophila* brain and delivering a certain amount of high frequency stimulation depending on their genotype, to induce seizures. The lowest possible intensity whereby seizures are elicited is known as the seizure threshold and if the flies fail to display seizures at the lowest possible intensity, the intensity is increased at 1V intervals until seizures are seen, with the fly being allowed to rest for several minutes in between each round of stimulation. Seizure activity in *Drosophila* is characterized by uncontrolled high frequency (> 100 Hz) neuronal firing and this can be most conveniently seen indirectly in the motor neurons that extensively innervate *Drosophila* thoracic muscle groups (Song et al., 2007; Kroll et al., 2015).

#### *Drosophila* model of refractory epilepsy

The multidrug resistance-associated protein 1 (MRP1) is a member of the Adenosine Triphosphate (ATP) binding cassette (ABC) superfamily and is a transporter of organic anions and drugs. As MRP1 is typically found in the blood brain barrier of humans, it likely functions as a protective mechanism to prevent the build-up of xenobiotics and drugs in the brain. However, an overexpression of MRP1 would also lead to a decrease in the extracellular concentration of AEDs in the brain and thus lead to refractory epilepsy. Unsurprisingly, MRP1 has been found to be overexpressed in the neurons and astrocytes of patients with refractory epilepsy (Sun et al., 2016).

*Drosophila* mutants with sodium ion channelopathies, such as those with *bss* phenotypes, have a lower seizure threshold as compared to normal flies and are thus more prone to seizure episodes (Parker et al., 2011; Howlett et al., 2013). Seizures in *bss* flies are typically very difficult to supress using either suppressor mutations or AEDs, making them potential models of refractory epilepsy (Howlett et al., 2013). It is also possible to model human refractory epilepsy in *Drosophila* by causing an overexpression of human MRP1 in bss mutant flies. Such flies are resistant to the seizure attenuation effects of phenytoin (acute and chronic application) as well as valproic acid (chronic application) (Bao et al., 2011). Thus, bss mutant flies which overexpress human MRP1 have the potential to become a model of human refractory epilepsy.

### Medicinal leech (*Hirudo verbena*)

With the emergence of non-mammalian models for use in research, the potential benefits of the medicinal leech for epilepsy research was evaluated. Despite its uncommon usage in epilepsy studies, medicinal leeches possess a relatively simple CNS in terms of neuron count. This is further supported by the similar physiological processes found in both leech and mammalian nervous systems in terms of neural circuitry and development. The maintenance and handling of leeches is also simpler and inexpensive as compared to mammalian models (Hahn and Burrell, 2015).

Only one study by Hahn and Burrell (2015) was conducted using the medicinal leech as an animal model in this review. They did this by first placing a single leech in a 100 ml beaker filled with 50 ml of pond water. PTZ (10 mM) was then added and the resulting motor behaviors observed in the medicinal leeches were categorized into normal (1 and 2) and abnormal (3 and 4). The level of motor behavior was further categorized into different classes: Type 1 – no activity, Type 2 – normal exploratory behavior with posterior sucker attached to beaker, Type 3 – abnormal exploratory behavior coupled with an inability to attach to the beaker, Type 4 – spontaneous twisting and tumbling behavior. The results reveal that spontaneous behavioral movement occurred during PTZ bath application as PTZ induced a concentration-dependent increase in motor activity within 5 min of treatment. The heightened motor activity was maintained for the period of PTZ treatment before reducing to baseline levels during washout. The PTZ treatment induced almost entirely abnormal behavior but did not cause any lethal effects. Pretreating the leeches with 1 mM of the AED phenytoin for 30 min prior to the PTZ treatment reduced the level of abnormal behavior by causing a reduction in motor activity, as well as affecting the behavior score.

### Planaria

Planaria are free-living non-parasitic flatworms of the phylum Platyhelminthes and possess a bilaterally symmetrical CNS composed of neurons similar to those of humans, a body plan common to all vertebrates and many invertebrates, as well as mammalian-like neurotransmitters (Rawls et al., 2009). Comparative genomic studies of invertebrate genomes such as the planarian *Schmidtea mediterranea*, with the human genome have identified gene and protein homologs, which portrays the fundamental principle that biochemical processes are comparable in simple and complex organisms (Ramakrishnan et al., 2013). Planarians have systems which correspond to all the major neurotransmission systems found in vertebrate brains, such as glutamate (excitatory) and GABA (inhibitory) (Ramakrishnan et al., 2013). Planarians also express the genes for at least two ionotropic glutamate receptor types, both of which have a high sequence similarity to neural specific genes isolated from both mice and humans. The effects of drugs acting on glutamatergic, serotoninergic, dopaminergic and cholinergic CNS neural transmission can also be examined in behavioral pharmacological studies involving planaria (Ramakrishnan and DeSaer, 2011). Invertebrate planaria also have the genes and neurotransmitters which correspond to the major inhibitory (GABA) and excitatory (glutamate) neurotransmission systems (Eriksson and Panula, 1994; Agata et al., 1998; Rawls et al., 2006). Even though these results suggest that glutamate-like receptors are important factors in planarian physiology, they are clearly not identical to mammalian glutamate receptors (Rawls et al., 2009). These differences in receptor homology and function may result in pharmacological effects that are not entirely the same across planarians and mammals. Nevertheless, planarian seizure models are easier to maintain and handle under laboratory conditions and are rather inexpensive in comparison with the other animal seizure models (Ramakrishnan and DeSaer, 2011), making planaria a fairly promising model for future research.

The usage of planaria models has been observed in three studies relating to epilepsy that were covered in this review.

#### Chemically induced seizures

Planaria exhibit sudden asynchronous convulsive movements which are very distinct from their normal locomotor activity, in proportion to the concentration of proconvulsant given (Ramakrishnan and DeSaer, 2011). *D. tigrina* exhibits screw-like hyperkinesis when exposed to 5 mM of picrotoxin. Planaria in the presence of low concentrations of (–)-nicotine (0.1 to 10 μM) display C-like and screw-like hyperkinesises whereas a more than 50 μM (–) of nicotine causes an increasing tendency to undergo longitudinal contraction, resulting in a walnut-like position. Once a planarian takes this walnut-like position, it essentially remains frozen in that position for a period of time (Ramakrishnan and DeSaer, 2011).

Nicotine, which is a cholinergic agonist, has also been found to induce hyperkinesia in planaria as neuronal nicotinic acetylcholine receptors (nAChR) play an excitatory role in the brain (Buttarelli et al., 2008). In addition, the molecular target for nicotine-induced seizures (α7 nAChR) was reported to be present in flatworms (Ribeiro et al., 2005). Planaria also exhibit seizure-like movements in nitrate and ammonia free tap water (Rawls et al., 2009). *Dugesia dorotocephala* has also been reported to display dose-dependent, seizure-like paroxysms when exposed to the excitatory neurotransmitters NMDA, α-amino-3-hydroxy-5-methylisoxazole-4-propionic acid (AMPA) and L-glutamate (Rawls et al., 2009).

### Roundworms (*Caenorhabditis elegans*)

*Caenorhabditis elegans* (*C. elegans*) is emerging as an important model for furthering insight into the cellular and molecular basis of neurological disorders (Dexter et al., 2012; Alexander et al., 2014). This microscopic nematode has several physiological similarities to mammals such as possessing ion channels, neurotransmitters and a conserved neuron morphology. *C. elegans* is small, inexpensive to maintain and has a relatively short 3-day generation time, all of which make large scale screening feasible (Risley et al., 2016). Seizures in *C. elegans* are typically induced via electrical shock or by increasing the ambient temperature (Pandey et al., 2010; Risley et al., 2016). Alternatively, convulsions in *C. elegans* can also be induced by mutations in the lis-1-allele (pnm-1), a defect in γ-aminobutyric acid (GABA) transmission (unc-25 and unc-49 mutants) or by PTZ and RNAi treatments in roundworms with depleted LIS1 pathway compounds (NUD-1, NUD-2 and DHC-1, CDK-5, CDKA-1) (Pandey et al., 2010). In the case of genetic changes, the resulting seizures which are induced in *C. elegans* are taken to be repeated contractions in either the dorsal or ventral directions (Pandey et al., 2010).

The usage of roundworm models has been observed in two studies relating to epilepsy that were covered in this review.

#### Electroshock assay for *C. elegans*

An electroshock assay similar to previously established convulsion models in fruit flies, was developed to quantitatively monitor paralysis duration and convulsions in *C. elegans* following electroshock. This method relies on the fact that young adult roundworms immediately exhibit paralysis with body stiffness and elongation following a brief three-second electric shock, though shocked animals resume normal movement after a short recovery period (Risley et al., 2016). As in mammals, the Maximal Electroshock Seizure Test (MEST) is the gold standard test for anticonvulsant activity (Risley et al., 2016).

#### High temperature assay for *C. elegans*

On the other hand, an increase in the surrounding temperature can cause abnormal bursts of neuronal cells which may be linked to seizures or convulsions (Pandey et al., 2010). Seizure index parameters were created by Pandey et al. (2010) as a way of quantify the convulsions in *C. elegans*. The seizure index is determined by placing the roundworms in a seizure promoting buffer (100 mM NaCl, 50 mM MgCl_2_) and then gradually increasing the temperature (26–28 ± 1°C) of the buffer using a variable intensity incandescent light source to generate heat. The seizures are ranked from 0 to 3, with 0 = no seizure or convulsion, 1(+) = two twitches in 10 s, 2(++) = two to five twitches in 10 s, 3(+++) = more than five twitches in 10 s or continuous twitching.

### Tadpole (*Xenopus laevis*)

Tadpoles have been utilized as an animal model in a small number of epilepsy studies. They possess many traits that allow for the detailed analysis of their developing nervous systems to study disorders and neural development (Pratt and Khakhalin, 2013). Similar to zebrafish, tadpoles have a similar neural circuitry as compared to other vertebrates and is homologous to other mammals, yet simpler in design. The epileptiform discharges recorded from the optic tectum of tadpoles show similarity to those recorded in zebrafish and rodents (Bell et al., 2011; Pratt and Khakhalin, 2013). Their transparency also conveniently allows for *in vivo* imaging of neuronal activity and development as well as synaptogenesis (Hewapathirane et al., 2008) Their small size increases cost effectiveness as tadpoles are low maintenance due to their basic requirements. Thus, their neurological traits coupled with simpler maintenance, reinforces their role as an animal model in epilepsy research and an effective alternative to other animal models.

The usage of tadpole models has been observed in two studies relating to epilepsy that were covered in this review.

#### Behavioral changes and testing of proconvulsants

A study relating to the *in vivo* imaging of seizure activity was conducted using a tadpole model. The tadpoles that were used in the study are transparent, which allows for the examination of seizure-induced effects on the developing brain. Five specific proconvulsants which include PTZ, picrotoxin (PTX), KA, pilocarpine and 4-aminopyridine (4-AP), were used to induce seizure behavior in the tadpoles (Hewapathirane et al., 2008; Bell et al., 2011). The first study discovered that all the proconvulsants tested can induce seizure behavior in tadpoles at different levels of severity. The following proconvulsants doses induced seizures within 20 min: PTZ (10–50 mM), PTX (1 mM), KA (0.25–1 mM), PILO (75 mM), 4-AP (0.5–2.5 mM). PTZ-induced seizures were given primary focus in ensuing experiments due to PTZ's dose range (10–50 mM) that rapidly causes severe seizures in the absence of toxic effects (Hewapathirane et al., 2008). Another study discusses the use of *Xenopus* tadpoles in examining the roles of polyamines in maintaining neural excitability. Bath application of 10 and 15 mM PTZ was used to induce seizures in tadpoles (Bell et al., 2011). Seizure behavior was observed after a few minutes exposed to PTZ and gradually increased, leading to loss of motor control (Bell et al., 2011). The seizure activity in both studies was characterized by directional control loss, immobility and C-shaped contractions.

#### *In Vivo* electrophysiological recordings

*Xenopus* tadpole models can also be used for electrophysiological recordings. In the first study, agar-immobilized tadpoles in a recording chamber were used to examine neuronal activity during proconvulsant-induced seizures, via extracellular field recordings (Hewapathirane et al., 2008). The recordings showed distinctive epileptiform discharges after exposure to different proconvulsants such as PTZ, PILO, 4-AP, and KA. The magnitude of epileptiform discharges gradually increases, eventually growing into high-amplitude spikes, in contrast to baseline recordings that are only comprised of sporadic low-amplitude spikes. The appearance of epileptiform discharges after proconvulsant washout varies based on the proconvulsant used. PILO (75 mM) induced spaced, sporadic high-amplitude epileptiform spiking (0.3 mV) whereas 4-AP (1 mM) caused sustained epileptiform discharges with a higher frequency. On the other hand, KA induced bursting in the form of fast-rhythmic spiking. Thus, it is possible to pinpoint the type of proconvulsant based on electrophysiological recordings using *Xenopus* tadpole models.

#### Analysis of seizure-related cell death

Two approaches were used to examine seizure-related cell death within the tadpole brain for the first study (Hewapathirane et al., 2008). One was the usage of fluorescein-based *in situ* terminal uridine deoxynucleotidyl transferase dUTP nick end labeling (TUNEL), which aimed to detect DNA fragmentation related to cell apoptosis. The second method utilized the *in vivo* incorporation of propidium iodide (PI). The results revealed that a low number of TUNEL-positive sand PI-positive cells (assays) were present throughout control brains, which indicated a reduction in continuous cell loss during normal development. Cellular apoptosis due to prolonged seizures (4 h) failed to increase when measured by TUNEL labeling over a span of 6 h−2 days post-seizure. PI labeled cells slightly increased, which is indicative of abnormal cell membrane permeability mostly linked with necrotic cell death and sometimes cell apoptosis (Hewapathirane et al., 2008).

### Zebrafish (*Danio rerio*)

Zebrafish have been acknowledged as one of the most widely-used animal models and have gained a reputation as an alternative to rodents and other animal models in epilepsy research over the last decade (Kundap et al., 2017). From a physiological perspective, they possess systems that are highly homologous to other vertebrates. The zebrafish genome has a 70% similarity to humans and zebrafish also possess complex anatomy and behavior which allows for the modeling of diseases (Cho et al., 2017). Both adult zebrafish and larvae can be used as animal models for epilepsy research, with larvae being the primary model in the majority of studies. Modification of gene and hence protein expression can be performed in zebrafish to produce genetically-modified zebrafish models by means of genetic intervention such as the injection of RNA, DNA and protein constructs during the early stages of embryo development (Berghmans et al., 2007). In contrast with rival models, zebrafish have lower maintenance costs and are capable of laying hundreds of eggs per week. Their complex behaviors and physiology allows for the modeling of diseases and mass drug screening (Cho et al., 2017). As a result, the increased utilization of zebrafish models in epilepsy research would present less economic issues to researchers.

Zebrafish have been used in 24 studies relating to epilepsy research that were covered in this review and are subdivided into the larval, adult and transgenic zebrafish subsections below.

### Larval zebrafish

#### Behavioral changes

The evaluation of behavioral changes in zebrafish larvae treated with proconvulsants plays a huge role in the understanding of epilepsy and seizures. As zebrafish larvae only become free-swimming at 3 days post-fertilization (dpf), they can only then be clearly evaluated for behavioral changes (Hortopan et al., 2010b). The observed behavioral changes are evaluated from the aspects of locomotor activity and thigmotaxis (Berghmans et al., 2007; Yang et al., 2017). Seizure score systems have been devised to categorize seizures based on the criteria above (Berghmans et al., 2007; Hortopan et al., 2010b; Afrikanova et al., 2013), to provide a more objective way of evaluating behavioral changes. All zebrafish larvae tend to display a similar pattern of seizure progression, with signs of increased agitation within seconds of seizure induction, followed by increased locomotor activity and thigmotaxis soon thereafter. This behavior soon progresses into “whirlpool-like” swimming behavior and rapid swimming. Brief convulsions and loss of posture soon follows, as the larvae enters the advanced stages of a seizure. The final stage of a seizure is marked by a decrease in the distance traveled by the larvae as they experienced brief convulsions and loss of posture (Berghmans et al., 2007; Tiedeken and Ramsdell, 2007; Hortopan et al., 2010b; Lee et al., 2012; Afrikanova et al., 2013). At higher concentrations of proconvulsant, zebrafish larvae expire due to neural damage (Berghmans et al., 2007). Zebrafish larvae can also be tracked on multi-well plates for high-throughput screening of behavioral changes (Yang et al., 2017). These behavioral changes are similar to that in adult zebrafish, with slight differences. Some notable differences include increased seizure onset times, possibly due to relatively immature respiratory and nervous systems in larvae resulting in a slower proconvulsant absorption rate. The degree of seizure activity is also largely dependent on the concentration and type of proconvulsant (Berghmans et al., 2007; Yang et al., 2017). As in adults, behavioral changes in proconvulsant treated zebrafish larvae can also be influenced by dark-light environments (Yang et al., 2017).

#### Utilization of proconvulsants

Zebrafish larvae provide a robust model that can be studied to understand the process of epileptogenesis through seizure induction using proconvulsants. In many of the studies, PTZ was primary proconvulsant used to induce seizures in zebrafish larvae. Other proconvulsants which have been used with zebrafish larvae include kainic acid, ginkgotoxin and picrotoxin (PTX) (Lee et al., 2012; Menezes et al., 2014; Yang et al., 2017). Proconvulsants are typically administered via bath application in multi-well plates, at different concentrations depending on the type of convulsant used. In majority of the studies covered, a 15–20 mM dose of PTZ was administered as these doses clearly produce all seizure scores in zebrafish larvae. Domoic acid (DA) can also be given to increase the seizure susceptibility of PTZ-treated zebrafish larvae by reducing the seizure threshold to produce an increase in locomotor activity (Lee et al., 2012).

PTX has also been used to induce seizures in other animal models (rodents and adult zebrafish) and causes significant increases in the locomotor activity of 5 dpf old zebrafish (Yang et al., 2017). Besides that, KA's efficacy in inducing seizure activity is dependent on the age of the zebrafish larvae as discovered by Menezes et al. (2014). These different results could be linked with state of the neural development in which the maturation of neurotransmitter systems influences the response to KA (Menezes et al., 2014). Similarly, ginkgotoxin at various concentrations (0.2–1 mM) causes hyperactive swimming in zebrafish larvae and also shows age dependent effects (Lee et al., 2012).

#### Screening of anti-convulsive compounds

Zebrafish larvae can also be used for the screening of anti-convulsive compounds by first pretreating them with the compound to be screened and then challenging them with a proconvulsant after a given habituation time. This screening method relies on the idea that if a compound is anti-convulsive, it should prevent or reduce the seizure behavior changes seen in proconvulsant treated zebrafish. Only microgram amounts of compound is required and can be quickly absorbed by zebrafish larvae through their gills, skin or gastrointestinal tract (Afrikanova et al., 2013). The small size of zebrafish larvae also allows it to thrive in small volumes, such as in the wells of a 96-well plate, which allows for the use of medium to high-throughput screening with an automated locomotor tracking system for the analysis of larval movement (Berghmans et al., 2007; Afrikanova et al., 2013). Prior to pretreatment, a toxicology evaluation may be performed to determine the maximum-tolerated concentrations (MTCs) of the drugs utilized, to avoid toxicity. This can be performed by using test compounds and observing the effects of AEDs on affected zebrafish for a period of 24 h (Barbalho et al., 2016).

#### Recording of brain electrical activity

Another aspect of epilepsy research in zebrafish larvae is the tracking of brain electrical activity using EEG and by the evaluation of field potential. By tracking brain electrical activity, the epileptiform discharges that occur in proconvulsant treated zebrafish larvae and hence the number of seizure events that occur, can also be monitored (Afrikanova et al., 2013; Hunyadi et al., 2017; Turrini et al., 2017). The measurement of local field potential (LFP) in zebrafish larvae can also be performed by placing glass electrodes into their optic tectum (Afrikanova et al., 2013). Besides that, genetically-encoded calcium indicators (GCaMP6) have also been utilized as an optical method to track neuronal activity in zebrafish larvae (Turrini et al., 2017). This method is based on fluorescent calcium indicators and relies on the fact that there is a correlation between concentration of intracellular calcium and spiking frequency. Thus, it can be used to analyse seizure activity and to map zebrafish larvae brains. The peak amplitude of any recorded spikes is directly proportional to the concentration of proconvulsant used to induce seizures in the zebrafish larvae (Turrini et al., 2017). In another study, both invasive LFP and non-invasive LFP were utilized in zebrafish larvae to record local field potential and to determine the number of seizure events by interpreting the number of epileptic discharges (Hunyadi et al., 2017).

### Adult zebrafish

#### Behavioral changes

The study of behavioral changes in adult zebrafish treated with proconvulsants is vital to epilepsy research. Behavior changes in adult zebrafish primarily involve its locomotor behavior. Aspects that are quantified in the evaluation of locomotor behavior include distance traveled, velocity, mobility, number of rotations and movement pattern of the zebrafish (Banote et al., 2013). Based on the severity of seizure activity, qualitative scoring systems were established to categorize zebrafish seizure activity into different scores (Pineda et al., 2011, 2013; Banote et al., 2013; Mussulini et al., 2013; Siebel et al., 2013; Gupta et al., 2014; Kundap et al., 2017; Choo et al., 2018). Initially, seizure activity begins with an increase in an adult zebrafish's swimming activity and is followed by short-burst, rapid swimming coupled with body jerking. Later stages of seizure activity are characterized by distinctive “whirl-pool” swimming behavior, clonic seizure-like behavior and loss of posture (Pineda et al., 2011; Mussulini et al., 2013). At a higher concentration of proconvulsant, zebrafish have a higher mortality rate due to the neural damage caused by epileptic seizures (Mussulini et al., 2013; Gupta et al., 2014). Severity of seizure activity and seizure latency are also largely dependent on the period of exposure to proconvulsant and the concentration of proconvulsant (Pineda et al., 2011; Mussulini et al., 2013; Siebel et al., 2013; Gupta et al., 2014). Other factors include gender differences, as demonstrated by one study which revealed that male zebrafish suffer more seizures than female zebrafish (Cho et al., 2017). In addition, the route of proconvulsant administration is also a factor as oral administration of proconvulsant elicits a seizure in adult zebrafish as quickly as bath application, but more weakly in terms of severity (Cho et al., 2017).

#### Utilization of proconvulsants

When challenged with proconvulsants, adult zebrafish express seizure behavior which can be observed and analyzed. PTZ was utilized as a proconvulsant for many adult zebrafish studies in this review (Pineda et al., 2011, 2013; Banote et al., 2013; Mussulini et al., 2013; Siebel et al., 2013; Gupta et al., 2014; Cho et al., 2017; Duy et al., 2017; Kundap et al., 2017). Other types of proconvulsants such as KA (Sierra et al., 2012) were used to a lesser extent. Proconvulsants are typically administered through the oral, intraperitoneal (Sierra et al., 2012; Banote et al., 2013; Kundap et al., 2017) and bath application (Pineda et al., 2011, 2013; Mussulini et al., 2013; Siebel et al., 2013; Gupta et al., 2014; Cho et al., 2017) routes. Each route of administration produces a similar behavioral response, with different seizure onset times at different concentrations. In the studies covered, PTZ injections were done using concentrations ranging from 170 to 220 mg/kg, while PTZ bath applications used concentrations ranging from 2 to 15 mM (Sierra et al., 2012). The results obtained from multiple studies showed that PTZ at a lower concentration (<7.5 mM) induced minimal seizure activity in the first 5 min and more severe seizure activity in the last 15 min, whereas PTZ at a higher concentration (>7.5 mM) induced severe seizures in the first 5 min (Pineda et al., 2011; Mussulini et al., 2013; Gupta et al., 2014). On the other hand, KA at a concentration of 6 mg/kg induces seizures in adult zebrafish, 2.5 min post-intraperitoneal injection, which is entirely different that the typical elicitation of seizures using PTZ in other studies (Sierra et al., 2012). Another study utilized caffeine as a proconvulsant with concentrations ranging from 1–30 μM. The results of the study revealed that the higher the concentration of caffeine applied, the lower the latency of seizure onset in zebrafish (Gupta et al., 2014).

#### Screening of anti-convulsive compounds

Much like their larval counterparts, adult zebrafish can and have been utilized for the screening of anti-epileptic compounds. The method of doing so is essentially the same as in zebrafish larvae, but a key difference is that adult zebrafish are larger in size and thus would be more difficult to screen at high throughputs. Another difference is a larger ranger of administration routes such as intraperitoneal injections (Kundap et al., 2017), which may be impossible or very challenging to perform in larvae.

#### Recording of brain electrical activity

The study of epilepsy in adult zebrafish also extends to the recording and evaluation of electrical activity within the brain. To track electrical activity within the brain of a zebrafish, an EEG may be used (Mussulini et al., 2013; Cho et al., 2017). Alternatively, an EEG equivalent for zebrafish would be the measurement of their cerebral field potential (Pineda et al., 2011, 2013; Banote et al., 2013). The long-term multichannel EEG utilizes a multichannel EEG electrode array for data acquisition. The two forms of seizure activity that can be recorded by an EEG are high-amplitude theta activities (5–7 Hz) which represent full-blown seizures similar as to TLE and low-frequency spike-wave activities which represent a an epilepsy discharge (2–3 Hz) (Cho et al., 2017). Similarly, other studies revealed high-amplitude sharp complexes in the measurement of the cephalic field potential in zebrafish, which is the result of seizure activity within the fish (Pineda et al., 2011, 2013; Banote et al., 2013). In addition, pre-treatment with AEDs resulted in a reduction in high-amplitude sharp complexes in PTZ-treated zebrafish, in a dose-dependent manner (Banote et al., 2013). It is further noted that continuous exposure to PTZ at 15 mM for 90 min resulted in a flat-line EEG, which is indicative of brain damage (Pineda et al., 2011; Mussulini et al., 2013).

### Transgenic zebrafish

Some studies have revealed that transgenic zebrafish models provide an efficient alternative to proconvulsants as they are more susceptible to seizures (Packer et al., 2016; Pena et al., 2017; Sourbron et al., 2017). Both transgenic adult and larvae models have been utilized in the studies covered.

#### Behavioral changes

Behavioral changes in transgenic zebrafish models have been analyzed in certain studies, usually those relating to seizure activity and motor behavior (Packer et al., 2016; Pena et al., 2017; Sourbron et al., 2017). A zebrafish model of *CLN3* disease or Batten's Disease was evaluated for seizure susceptibility, which revealed the first sign of motor abnormality at 36 h post-fertilization (hpf) whereby the *CLN3* ATG morphants displayed higher movement activity and a higher number of tail flicks, both of which are indicative of seizure activity (Packer et al., 2016). Another study discussed the prospects of having a mutant *scn1a* gene be expressed in zebrafish as high levels of locomotor activity were observed in zebrafish scn1a mutants (Sourbron et al., 2017). The last study conducted presented *aldh7a1* mutant zebrafish, with pyridoxine-dependent epilepsy (PDE) that showed signs of hyperactivity characterized by “whirlpool” swimming and loss of posture by 10 dpf when stimulated with light (Pena et al., 2017).

#### Seizure susceptibility of transgenic models

The seizure susceptibility of transgenic models differs from each other to a certain degree and is influenced by external factors such as the presence of light and the age of the animal. For example, *cln3* ATG mutants experience motor abnormalities at 36 hpf while aldh7a1 mutants experience motor abnormalities at 10 dpf. The *aldh7a1* mutant is also affected by light stimuli, which appears to worsen the motor abnormality, causing “whirlpool” swimming activity and eventually loss of posture as it increases seizure susceptibility (Packer et al., 2016; Pena et al., 2017). Another study discusses the accumulation of piperideine 6-carboxylate (P6C), which can eventually lead to an increased seizure occurrence in affected zebrafish (Pena et al., 2017). An *aldh7a1* mutation refers to PDE in which the seizure activity is dependent on levels of pyridoxine (Pyr). Aldh7a1 mutant larvae that were treated with Pyr have significantly suppressed seizure activity and daily treatment successfully prolonged their lives. The immediate withdrawal of Pyr treatment resulted in seizure activity and eventual death (Pena et al., 2017).

#### Electrographic component of induced seizures within transgenic models

The study of transgenic zebrafish models also extends to the tracking and evaluation of epileptiform electrographic activity. Once again, EEG was used for the recording of brain electrical activity. The field potential recordings acquired from both *aldh7a1* mutants when stimulated with light, showed that the *aldh7a1* mutant was suffering from spontaneous seizure activity which was marked by sudden bursts of high amplitude, long duration waves that were similar in appearance to the spontaneous seizure activity in other animal models. In comparison, wild type zebrafish had normal electric activity characterized by the absence of high amplitude waves (Pena et al., 2017). Similar results were also observed in a surface EEG conducted in using *cln3* ATG MO morphants injected with p53 MO and ng *cln3* ATG MO. Significantly higher amplitude waves with a frequency of 2–4 Hz was recorded, which reflects epileptiform activity (Packer et al., 2016). Sporadic multi-spike bursts which were similar to ictal-like waveforms were also recorded in 93% of mib^hi904^ zebrafish following exposure to 15 mM of PTZ (Hortopan et al., 2010a).

## Discussion

Research using non-mammalian models of epilepsy offers substantial advantages for identifying and studying several seizure parameters including behavior, different types of cellular activities, electrophysiological changes and the genes involved in regulating seizures (Table 2). Though there are many insults that may be used to trigger seizure like behavior in the non-mammalian models of epilepsy, the behavioral changes that result are typically heightened motor activity. While these seizure behaviors can be measured qualitatively (the absence or presence of seizures), seizure scoring systems for the quantitative measurement of seizure behavior have also been designed for certain non-mammalian models such as zebrafish (Kundap et al., 2017) and roundworms (Pandey et al., 2010). Having a more finely grained method of scoring seizures could enable anti-epileptic compound screens to also detect compounds with less than ideal anti-epileptic activity in its unmodified form but could potentially become more potent after chemical modification. This is useful as even the first line AEDs being used today may not completely or permanently supress seizures at the more conservative doses usually preferred (Xia et al., 2017). However, care should be taken as the method of inducing seizures and the route of administration could potentially affect the animal in ways that may be directly observable (type and onset of behavioral changes) or otherwise (changes in neuronal activity).

**Table 2:** Characteristics, advantages, disadvantages, proconvulsants used with and the seizure behavior of the different non-mammalian models of epilepsy in this study.

**Models**	**Features**	**Proconvulsant**	**Behavior**	**Uses/advantage**	**Limitations**
Fruit fly (*Drosophila melanogaster*)	•High breeding rate •Low maintenance cost •Large chromosomes that are easy to manipulate •Quick and inexpensive genetic manipulation is possible •Completely sequenced genome •Relatively high genetic similarity to humans	•Pentylenetetrazole •Valproic acid •Picrotoxin •4-aminopyridine •Electrical stimulation •Heat-induced seizure •Light stimulus (Transgenic models) •Mechanical shock	Loss of posture with leg shaking, abdominal muscle contractions and wing flapping	•Growth can be accelerated by heat •Short generation times •Assessment of seizure-induced behavior •Testing of proconvulsants •Testing of anti-epileptic drugs •Screening of potential anti-epileptic compounds	•Anatomy of flies and humans are very different •Complex behavior is difficult to measure •Effect of drugs is less predictable due to the differences in body systems
Medicinal Leech (*Hirudo verbana*)	•Simple central nervous system in terms of number of neurons •Similar physiological •processes of nervous system to mammalian nervous system •Seizure-like activity in leeches are applicable to human CNS through physiological processes •Low maintenance cost and easy handling	Pentylenetetrazole	Spontaneous twisting and tumbling behavior with inability to attach to beaker	•Assessment of seizure-induced behavior •Testing of proconvulsants •Testing of anti-epileptic drugs	Epilepsy studies involving medicinal leeches are lacking
Planaria	•Many planarian proteins are significantly similar to human proteins •RNA interference can be efficiently carried out by feeding, injecting or soaking planaria with solutions of double-stranded RNA •Planarians possess genes and neurotransmitters which correspond to all the major neurotransmission systems found in vertebrate brains •Low maintenance cost and easy handling	•N-methyl-D-aspartate •Picrotoxin •Nicotine •Semicarbazide •Glutamate	Increasing number of sudden asynchronous convulsive movements	•Assessment of seizure-induced behavior •Testing of proconvulsants •Testing of anti-epileptic drugs •Planaria can regenerate lost tissue or limbs •Modeling addiction behaviors	Epilepsy studies involving planaria are lacking The glutamate-like receptors in planaria are not identical to those in mammals
Roundworm (*Caenorhabditis elegans*)	•The hermaphrodite roundworms can self-fertilize •Complete gene sequenced •Can be grown cheaply and in large numbers on plates containing bacteria •Roundworms are small in size and produce over 1000 eggs per day, with a short life cycle of just 2 weeks •Many roundworm genes have human counterparts •Low maintenance cost and easy handling	•Pentylenetetrazole •Electrical stimulation •Heat-induced seizure	Multiple contractions while moving in the same direction	•Assessment of seizure-induced behavior •Testing of proconvulsants •Testing of anti-epileptic drugs •Screening of potential anti-epileptic compounds •Producing genetic mutants to model certain human diseases	Epilepsy studies involving roundworms are lacking Lacks many defined organs/tissues
Tadpole (*Xenopus laevis*)	•Similar neural circuitry to other vertebrates •Genetically homologous to other mammals •Similar to zebrafish in terms of recording epileptiform discharges •Low maintenance cost and easy handling	•Pentylenetetrazole •Kainic acid •Picrotoxin •Pilocarpine •4-aminopyridine	Directional loss, immobility and C-shaped contractions.	•Assessment of behavior of seizure-induced tadpole •Measurement of neural activity in brain during seizures •Testing of proconvulsants •Application of electrophysiological recordings •Examination of seizure-related cell death	Epilepsy studies involving tadpoles are lacking
Zebrafish (*Danio rerio*)	•Both larvae and adult zebrafish can be used as models •Mass-breeding capabilities up to hundreds of eggs weekly •Rapid growth and development rate •70% genetic similarity with human •84% of human diseases can manifest in zebrafish •Low maintenance cost (<0.01$ per day) and easy handling •Can be genetically modified in early embryo development	•Pentylenetetrazole •Kainic acid •Ginkgotoxin •Picrotoxin •Caffeine •Light stimulus (Transgenic models)	**Larvae** – Thigmotaxis, rapid and “whirlpool” swimming followed by loss of posture **Adults** – Short-burst rapid swimming followed by distinctive “whirlpool” swimming and loss of posture	•High-throughput screening for drugs, specifically antiepileptic drugs •Assessment of behavior of seizure-induced fish •Analysis of epileptogenesis process •Measurement of neural activity in brain during seizures •Testing of various proconvulsants and antiepileptic drugs •Producing genetic mutants to model certain human diseases	High mortality Sensitivity to proconvulsants differ in each fish tested Lack of reliable EEG equivalents to record brain electrical activity in adult fish

Although the rodent seizure models continue to serve as the foundation for basic and translational epilepsy research, unconventional vertebrate (zebrafish) and invertebrate (fruit fly and roundworm) models are proving to have greater potential for analyzing the epilepsy phenotype as they are genetically tractable organisms (Baraban, 2007). As much regarding epilepsy is yet unknown, the ease of genetically modifying such organisms coupled with short generation times would greatly aid in identifying additional molecules, gene or signaling mechanisms which affect epileptogensis and epileptic behavior to better understand epilepsy. One possible use of genetically alterations is to produce mutant non-mammalian models of epilepsy which have different seizure thresholds. This would also aid understanding of the epilepsy phenotype by allowing researchers to mimic the clinical conditions of a wide variety of different epilepsy types. One day, it could even become possible to insert genes which confer seizure resistance into humans in the form of gene therapy to help ameliorate epilepsy, though there are many hurdles to overcome before that day arrives.

While the similarity of certain non-mammalian seizure like behaviors to human seizures (Lucey et al., 2015) is a great boon, the dissimilarity of these non-mammalian models to humans also ironically makes them ideal models. As an example, the non-mammalian models covered in this review typically have a relatively simpler CNS, yet retain much of the functionality and characteristics of their more complicated mammalian counterparts such as the major neurotransmission systems (Ramakrishnan et al., 2013) as well as neural circuitry and development (Hahn and Burrell, 2015). As the non-mammalian models covered in this review are relatively small, they can be easily and cheaply maintained in large numbers with little logistical and financial difficulties. Their small body size would also reduce the amount of chemicals such as proconvulsants and novel compounds that are needed for experimentation or screening, if they are available in limited quantities or are prohibitively expensive. In addition to their small size, the high breeding rate of some non-mammalian models also facilitates the use of high-throughput screening (Yang et al., 2017). When taken individually, it becomes clear that each non-mammalian model in this review has different characteristics, advantages, limitations and seizure behavior in response to different methods of inducing seizures.

The seizures induced in animal models typically mimic either human generalized or partial seizures. As mentioned previously, different seizure induction methods can be used to induced different seizures types. For example, MES can induce generalized tonic-clonic seizures whereas PTZ can induce non-convulsive generalized seizures (Löscher, 2011). That being said, it is worth noting that these seizure induction methods actually have poor face validity as the defining feature of epilepsy are the spontaneous recurrent seizures (Mussulini et al., 2013). However, the seizures induced by certain chemoconvulsants such as PTZ only cause transient acute seizures rather than true epilepsy. Nevertheless, the National Institute of Neurological Diseases and Stroke (NIH) actually recommends the use of PTZ and MES to induce acute seizures during the initial screening process (National Institute of Neurological Disorders and Stroke, 2013). This is likely an attempt to save both time and resources as although chronic seizure models using the proconvulsants kainic acid and pilocarpine which more closely mimic true human epilepsy exist, they require a significant investment in time and resources to carry out. That being said, the construct and predictive validity of acute seizure models is not entirely zero as compounds which show great anticonvulsive potential in such models would be more likely to also display that potential in true chronic models of epilepsy. The articles found in this review seem to support this belief, such as that by Kundap et al. (2017) who found that currently available anti-epileptic drugs which work in humans, also counteract PTZ induced seizures. It is also worth noting that although anti-epileptic drugs are so termed, they are actually anti-convulsants which only provide symptomatic treatment rather than treat the underlying cause of epilepsy.

With all the advantages of non-mammalian models over rodent models, one might wonder why rodent models remain so prolific? One of the reasons could be that rodents have been an established model since the days of old for their similarities to humans both anatomically and genetically as well as having a short reproductive cycle (Butterweck and Nahrstedt, 2012). Another reason for the widespread use of rodents, especially in the case of pharmacological safety and pharmacokinetic studies of potential novel drugs, is simple regulatory in nature. This is because drug regulatory agencies typically require the use of at least two mammalian species, including one non-rodent species, prior to the authorization of human trials (Atanasov et al., 2015). While the practice of using rodent models for epilepsy research no doubt arose from the cumulative work of our predecessors, perhaps it is time for a paradigm shift in the form of non-mammalian models. Rather than conducting preliminary studies using non-mammalian models before committing to rodent models for promising compounds, perhaps future pre-clinical epilepsy studies could be done solely in non-mammalian models and proceed directly to human trials if successful. This is supported by the findings that the disease-related genes in non-mammals such as zebrafish (84%) and drosophila (75%) have human orthologs (Pandey and Nichols, 2011; Kalueff et al., 2014). This coupled together with the human seizure like behavior and a higher effectiveness of non-mammalian models of epilepsy could 1 day allow them to replace rodent models of epilepsy instead of merely supplanting them. It is hoped that the information on the importance and usage of non-mammalian models in studies concerning epilepsy provided by this review will serve as a stepping stone for future research into the field of epilepsy and also the screening of anti-convulsive compounds.

## Conclusion

In conclusion, non-mammalian models of epilepsy covered in this review have already shown great promise in the field of epilepsy research and the animals themselves offer many advantages over the typical mammalian rodent models of epilepsy. It is hoped that future research will take advantage of all these non-mammalian models of epilepsy as the tadpole, planarian, roundworms and medicinal leech models appear to be underutilized as compared to zebrafish and fruit flies, in terms of publication numbers. These models should not be discounted as researchers will need every possible tool at their disposal in the difficult quest to unravel the enigma that is epilepsy and possibly even discover a treatment for it 1 day.

## Author contributions

MJ and BC have joint first authorship for this publication. MJ and YL were responsible for the searching and screening of articles for this review. MJ, BC, and YL were responsible for the writing of the manuscript. MS was responsible for conceptualizing and revising the manuscript. YK was also involved in conceptualizing, proofreading and diagram preparation. All authors gave their final approval for the submission of the manuscript.

### Conflict of interest statement

The authors declare that the research was conducted in the absence of any commercial or financial relationships that could be construed as a potential conflict of interest.
